# Image-guided versus blind corticosteroid injections in adults with shoulder pain: A systematic review

**DOI:** 10.1186/1471-2474-12-137

**Published:** 2011-06-25

**Authors:** Edmund Soh, Wenyun Li, Keh Oon Ong, Wen Chen, Dianne Bautista

**Affiliations:** 1 Department of Diagnostic Radiology, Singapore General Hospital, Singapore; 2 Department of Biostatistics, Singapore Clinical Research Institute, Singapore; 3 Center for Quantitative Medicine, Office of Clinical Sciences, Duke-National University of Singapore Graduate Medical School, Singapore; 4 Hitachi Aloka Ultrasound Division, Hitachi Medical Systems (S) Pte Ltd, Singapore

## Abstract

**Background:**

Corticosteroid injections can be performed blind (landmark-guided) or with image guidance, and this may account for variable clinical outcomes. The objective of this study was to assess the effectiveness and safety of image-guided versus blind corticosteroid injections in improving pain and function among adults with shoulder pain.

**Methods:**

MEDLINE, the Cochrane Controlled Trials Register and EMBASE were searched to May 2010. Additional studies were identified by searching bibliographies of shortlisted articles. Search items included blind, landmark, anatomical, clinical exam, image-guided, ultrasound, fluoroscopy, steroid injection, frozen shoulder, random allocation, randomized controlled trial (RCT) and clinical trial.

Randomized controlled studies comparing image-guided versus blind (landmark-guided) corticosteroid shoulder injections that examined pain, function and/or adverse events were included. Independent extraction was done by two authors using a form with pre-specified data fields, including risk of bias appraisal. Conflicts were resolved by discussion. The decision to pool data was based on assessment of clinical design homogeneity. When warranted, studies were pooled under a random-effects model.

**Results:**

Two RCTs for pain, function and adverse events (n = 101) met eligibility criteria. No serious threats to validity were found. Both trials compared ultrasound-guided versus landmark-guided injections and were judged similar in clinical design. Low to moderate heterogeneity was observed: shoulder pain I^2 ^= 60%, function I^2 ^= 22%. A meta-analysis demonstrated greater improvement with ultrasound-guided injections at 6 weeks after injection in both pain (mean difference = 2.23 [95% CI: 1.27, 3.18]), as assessed with a 0 to 10 visual analogue scale, and shoulder function (standardised mean difference = 1.09 [95% CI: 0.61, 1.57]) as assessed with shoulder function scores. Although more adverse events (all mild) were reported with landmark-guided injections, the difference was not statistically significant (risk ratio = 0.20 [95% CI: 0.04, 1.13]).

This review was only based on two moderate-sized trials. Blinding of patients was not performed in both trials, causing some risk of bias in outcome assessment since primary endpoints were wholly or partially patient-reported.

**Conclusion:**

There is a paucity of RCTs on image-guided versus landmark-guided corticosteroid shoulder injections examining pain, function and adverse events. In this review, patients who underwent image-guided (ultrasound) injections had statistically significant greater improvement in shoulder pain and function at 6 weeks after injection. Image-guided (ultrasound) corticosteroid injections potentially offer a significantly greater clinical improvement over blind (landmark-guided) injections in adults with shoulder pain. However, this apparent benefit requires confirmation from further studies (adequately-powered and well-executed RCTs).

## Background

Shoulder pain is common in the general population. Corticosteroid injections are widely used to treat shoulder pain irrespective of the underlying aetiology (e.g. rotator cuff disease, bursitis, adhesive capsulitis, etc.). The injections can be performed "blind" (via anatomical landmarks to guide placement of the needle) or with image guidance (usually ultrasound with visualisation of the needle tip at the target site) [1,2]. Studies have demonstrated that needle placement is more accurate with image guidance [3]. However, it is more controversial whether accuracy of needle placement has a significant impact on clinical outcome. Some studies have demonstrated improvement in shoulder symptoms irrespective of whether the needle was in the targeted structure or not [4]. Others have reported improved clinical outcome with image-guided injections [3].

These conflicting results may have come about from different study designs. A systematic review assessing whether there is a significant difference in clinical outcome between blind (landmark-guided) and image-guided injections is required to assess the best-available evidence.

## Objective

To assess the effectiveness and safety of image-guided versus blind (landmark-guided) corticosteroid injections in adults with shoulder pain. Outcome measures for effectiveness included change in pain and function scores. Safety was assessed by documentation of adverse effects.

## Methods

### Eligibility criteria

We considered randomized controlled trials of image-guided versus blind (landmark-guided) corticosteroid shoulder injections in adults 18 years and above with shoulder pain due to soft tissue disorders. Specific exclusions were shoulder pain due to osseous pathology (e.g. osteoarthritis, osteonecrosis), duration of shoulder pain less than three weeks, inflammatory joint disease, previous trauma in the shoulder region, previous physiotherapy and previous local steroid injection. Only single corticosteroid injections over time were included. Anatomical target sites included the glenohumeral joint, the subacromial space and specific tendon sheaths. All corticosteroid preparations of various volumes and types were included. All image-guided techniques were considered including ultrasound and fluoroscopy. The primary outcome measures were pain as assessed by the visual analogue scale (VAS) and shoulder function by any validated scale such as the Constant Score. Safety, a secondary outcome, was evaluated by the frequency of adverse events. Effectiveness was assessed by the change in pain and function scores evaluated at baseline and the final assessment period.

### Information Sources and Search

Studies were identified by searching electronic databases and the bibliographies of shortlisted articles. MEDLINE, the Cochrane Controlled Trials Register and EMBASE were searched in all languages to May 2010. A limited update literature search was made from 30 May 2010 to 14 March 2011. Key search terms were *blind*, *landmark*, *anatomical*, *clinical exam*, *image-guided*, *ultrasound*, *fluoroscopy*, *steroid injection*, *frozen shoulder*, *random allocation*, *randomized controlled trial (RCT) *and *clinical trial*.

### Study selection

Three authors (ES, WL, and DB) assessed the titles and abstracts from the electronic search for eligibility. The full-text article of shortlisted studies were then retrieved and further assessed. All review authors decided on study inclusion.

### Data collection process

Two authors (DB, WL) independently extracted characteristics of included studies using a form with pre-specific data fields. The following characteristics were sought: study design, participants (eligibility criteria) and setting, interventions (type of injection, corticosteroid preparation, person delivering the injection), length of follow-up, assessment periods, and outcomes. Primary study authors were contacted by email for additional details when required.

### Risk of bias appraisal

The same authors (DB, WL) also appraised studies independently for internal validity by examining sequence generation, allocation concealment (both at the study level), blinding of patients, investigators and outcome assessors, incomplete data reporting, selective outcome reporting (all at the outcome level) and other biases following Cochrane review methods for interventions. Differences were resolved by referring to the original article and/or by discussion with a third party (ES). Only trials with low or unclear risk of bias in sequence generation and allocation concealment were included in the meta-analyses. Data were entered and analyzed in the Revman Review Manager V5 software [5].

### Summary measures

For pain and shoulder function scores, treatment effects were summarized by the mean difference (MD) and standardized mean difference (SMD) respectively. For summarizing safety (frequency of adverse events), the risk ratio (RR) was used.

### Synthesis of results

Heterogeneity in clinical design was ascertained by examination of the table of characteristics of included studies. Statistical heterogeneity was quantified using the I^2 ^statistic and the chi-square-based test. When warranted, pooling was made under a random effects model, in view of anticipated differences in clinical design [6].

## Results

**Figure 1:**
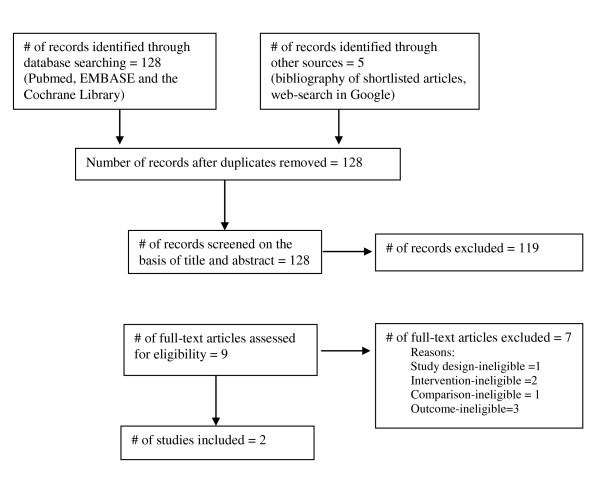
**Flow diagram of study selection**.

The search yielded nine potential studies (Figure 1), of which two satisfied inclusion criteria [7,8]. The seven excluded studies are listed in references [9-15]. Detailed patient characteristics and aspects of clinical design of the two included studies are provided in Additional File 1. The two trials were similar in clinical design (total patients = 101, image-guided = 51 and landmark-guided = 50). Both compared ultrasound-guided versus landmark-guided injections. With only two studies available, estimates of statistical heterogeneity were deemed imprecise. The estimated proportion of between-study variability (I^2^) was 61% for shoulder pain and 22% for shoulder function. Based on the chi-square test, no significant statistical heterogeneity was found in all outcomes but this is likely due to low statistical power.

**Figure 2:**
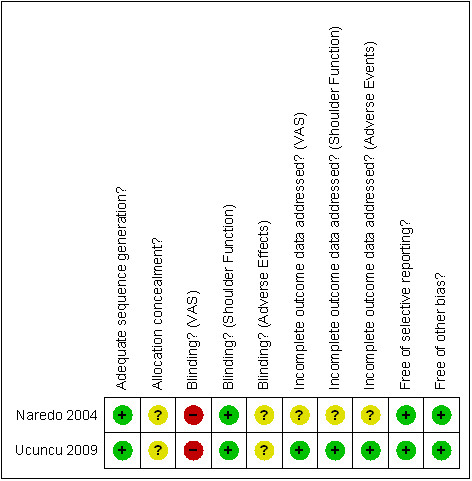
**Risk of bias assessments (Cochrane Collaboration) of included studies**. + indicates a low risk of bias; - indicates a high risk of bias;? indicates an unclear risk of bias.

Shoulder function was assessed by the Constant score or the Shoulder Function Assessment scale. Shoulder pain was assessed by VAS. Risk of bias assessments are outlined in Figure 2. Patients were not blinded to the injection technique and this may have resulted in some bias particularly for purely subjective assessments such as VAS (a self-assessment scale). The risk of bias for shoulder function assessment in terms of blinding was judged to be of low risk in both studies. Some risk of bias assessments were judged unclear as no specific details in the studies could be obtained from the study authors for the relevant assessment. No serious threats to validity were found.

**Figure 3:**
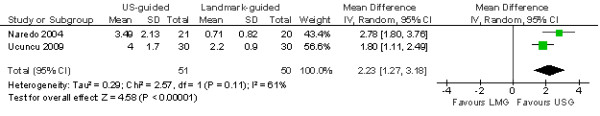
**Forest plot of shoulder pain (VAS)**.

**Figure 4:**
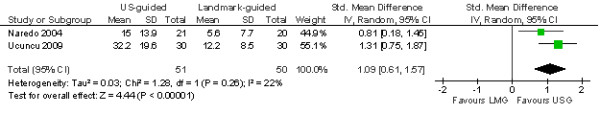
**Forest plot of shoulder function**.

**Figure 5:**
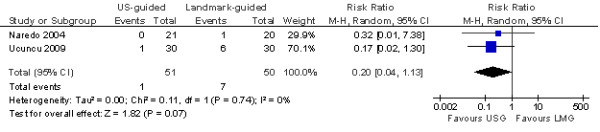
**Forest plot of number of adverse events**.

Using a random effects model, pooling of data from both trials demonstrated statistically significant greater improvement with ultrasound-guided injections at 6 weeks after injection in both pain (mean difference = 2.23 [95% CI: 1.27, 3.18]), as assessed with a 0 to 10 visual analogue scale, and shoulder function (standardised mean difference = 1.09 [95% CI: 0.61, 1.57]) as assessed with shoulder function scores (Figures 3 and 4). More adverse effects (all mild) were also reported with blind injections though the difference was not statistically significant (risk ratio = 0.20 [95% CI: 0.04, 1.13]; Figure 5). Adverse events included slight increase in pain and skin peeling post-injection.

## Discussion

Corticosteroid injections are commonly used to treat shoulder pain although studies assessing the efficacy of such injections have given conflicting results [16,17]. Conclusions of systematic reviews have been limited by small sample sizes and variable methodological quality and heterogeneity. This current review sought to assess the effectiveness and safety of image-guided versus blind corticosteroid injections. To our knowledge, such a review has not been published before. Patients who underwent image-guided (ultrasound) injections had statistically significant greater improvement in shoulder pain and function at 6 weeks after injection, and also had less adverse events. These findings would suggest that ultrasound-guided corticosteroid injections are more beneficial than blind injections. The results should be interpreted with some caution due to the limited number of studies and small sample sizes of the two included studies.

Any beneficial effect of steroid injections is likely due to its anti-inflammatory effect. Inaccurate placement of steroid may result in a partial response due to further diffusion of steroid away from its target site. Henkus et al. reported that 62.5 to 76% of subacromial injections were accurately placed when given blind, the intended target being the subacromial bursa. Injections isolated to the subacromial bursa resulted in significantly decreased pain and improved functional scores, whereas injection of other structures resulted in increased pain scores [18]. Eustace et al. reported 29% (4 out of 14) of subacromial and 42% (10 out of 24) of glenohumeral joint injections were accurately placed when given blind. There was also a positive correlation between clinical outcome and accurately placed injections [19].

Ultrasound is a safe and accurate technique for guiding aspiration and infiltration that ensures correct placement of the needle and delivery of the drug. Ultrasound-guided injections allow direct visualisation of the needle in real-time as it pierces the skin to entering the target site. Ultrasound scanning can also be performed immediately after injections to visualise the location of the steroid deposit which appears as echogenic foci or lines, with or without acoustic shadowing [20]. Imaging in general (including ultrasound) requires more resources (skilled manpower, imaging equipment, etc.), and at our institution, imposes a higher financial expense to the patient. However, appropriate use of imaging is better answered with a cost-effectiveness study to assess whether significant healthcare savings can be achieved overall.

One limitation of the reviewed studies is that the participants were not blinded for treatment group and this may have resulted in some bias favouring ultrasound-guided injections, particularly with self-reporting assessments. However, successful blinding of participants to fulfil a double-blind study is difficult to achieve in practice. Another limitation is the small number of high-quality studies available for review and the small sample sizes in the available studies. In addition, ultrasound-guided injections in the included studies were performed by non-radiologists. It is debatable whether there will be a significant difference if the image-guided injections were performed by radiologists, who arguably do more image-guided injections than other healthcare professionals, certainly at least in our institution. We would like to see more studies comparing blind versus image-guided injections, including the former performed by experienced clinicians and the latter performed by experienced radiologists.

## Conclusion

Image-guided (ultrasound) corticosteroid injections potentially offer a significantly greater clinical improvement over blind injections in adults with shoulder pain. The results should be interpreted with some caution due to the limited number of studies and small sample sizes available for review. More adequately-powered and well-executed RCTs are required.

## Competing interests

W Chen is a salaried employee at Hitachi Systems (S) Pte Ltd, a manufacturer of medical equipment including ultrasound machines. The authors declare that there are no other competing interests. Hitachi Medical Systems (S) Pte Ltd did not finance the manuscript and was not involved in manuscript preparation.

## Authors' contributions

All authors contributed to study design. WL and DB extracted data, appraised for risk of bias and performed statistical analysis. All authors read and approved the final manuscript.

## Pre-publication history

The pre-publication history for this paper can be accessed here:

http://www.biomedcentral.com/1471-2474/12/137/prepub

## Supplementary Material

Additional files 1 **Characteristics of included studies**. Detailed patient characteristics and aspects of clinical design of the two included studies. Click here for file
