# Supplementary material for: Image-guided versus blind corticosteroid injections in adults with shoulder pain: A systematic review
Source: BMC Musculoskelet Disord. 2011 Jun 25;12:137. doi: 10.1186/1471-2474-12-137 (PMC3135580; doi:10.1186/1471-2474-12-137)
Supplement: Additional files 1 — Characteristics of included studies. Detailed patient characteristics and aspects of clinical design of the two included studies. [file 1471-2474-12-137-S1.DOC]

**Additional File 1** Characteristics of included studies

|  |  | **Naredo 2004 (RCT)** |  | **Ucuncu 2009 (RCT)** |
| --- | --- | --- | --- | --- |
| **Eligibility Criteria** | **Included** | First flare of shoulder pain of periarticular origin e.g., impingement syndrome, rotator cuff lesions, SASD bursitis, and/or biceps tendon abnormalities, established by clinical history and examination, ≥ 1 month duration without response to NSAID |  | Soft tissue disorders e.g. acromioclavicular degeneration, rotator cuff lesions (rupture, partial rupture, tendinosis, impingement, calcification), fluid accumulation in biceps tendon, partial rupture in the biceps tendon and bursitis (SASD), ≥ 1 month duration without response to NSAID for ≥ 1 month |
|  | **Excluded** | Chronic inflammatory arthritis, glenohumeral osteoarthritis, previous trauma, fracture, bone tumor, osteonecrosis, other bone conditions as established by plain radiographs; previous physiotherapy, previous local steroid injection in same shoulder, previous steroid injection in any musculoskeletal location in 3 months prior to the study |  | chronic inflammatory arthritis, diabetes mellitus, previous major trauma in shoulder area, pain in both shoulders, fracture, glenohumeral osteoarthritis, bone tumor, osteonecrosis, other bone conditions, previous physiotherapy, or previous local steroid injection, established by at least one of the following: patient history, physical exam, laboratory tests (hemogram, WBC, routine biochemistry, sedimentation, and C-reactive protein), and plain radiographs |
| **Setting** |  | Severo Ochoa Hospital, Madrid, Spain (university hospital) |  | Farabi Hospital, Trabzon, Turkey (university hospital) |
| **Interventions** | **LMG** | **N=20**  Injection of 20 mg triamcinolone using a 21-gauge needle.  Lateral entry approach to subacromial region; needle inserted under anterolateral aspect of acromion process, through deltoid muscle and directed medially and slightly anterior to SASD bursa |  | **N=30**  1 mL of 40 mg triamcinolone and 1 mL of 1% lidocaine, for a total of 2 mL  Lateral entry approach to subacromial region (no further details) |
|  | **USG** | **N=21**  Injection of 20 mg triamcinolone using a 21-gauge needle.  Transducer in one hand and syringe in the other; needle placed under probe with route visualized real time by US as hyper-reflective line from skin to target; when increased fluid was detected, injection was into the SASD bursa or biceps tendon sheath; when effusion in both, injection into SASD bursa; peri- and intra-lesional injection done when rotator cuff calcification was present |  | **N=30**  1 mL of 40 mg triamcinolone and 1 mL of 1% lidocaine, for a total of 2 mL  Injection directed to target beneath probe by imaging a hyper-reflective line; administered peri-lesionally and intralesionally (no further details) |
| **Outcomes, follow-up and assessment** | **Primary** | Visual Analogue Scale (0-100 mm) for pain and Shoulder Function Assessment scale (0-70) evaluated on admission and six weeks post-injection, and post-injection adverse effects |  | Visual Analogue Scale (0-10) for pain and Constant Score for function assessment (0-100), active and passive ROM with goniometric evaluation, assessed on admission and six weeks post-injection, and post-injection adverse effects |
|  | **Secondary** | Presence of nocturnal pain, intake of NSAID, active and passive ROM; accuracy of injection placement; proportion with 50% improvement in VAS and SFA scores calculated from baseline |  |  |
|  | **Remark** | No physiotherapy allowed during follow-up; patients with loss of ROM performed home-based pendulum and shoulder abduction exercises; no limits on use of shoulder to the extent tolerable nor on NSAID use |  | No physiotherapy allowed during follow-up; patients with loss of ROM performed home-based pendulum and shoulder abduction exercises; no limits on use of shoulder to the extent tolerable nor on NSAID use |

**Additional File 1** Characteristics of included studies (continued)

|  | **Baseline Characteristics*** |  |  |
| --- | --- | --- | --- |
|  |  | **LMG** | **UMG** |
| **Naredo 2004** |  |  |  |
|  | **Number** | 20 | 21 |
|  | **Age** | 51.9 (13.8) | 52.9 (11.0) |
|  | **% Female** | 12 (60.0) | 15 (71.0) |
|  | **Duration of symptoms** | 10.2 (14.7) | 11.9 (14.6) |
|  | **Affected shoulder** | R-11 (55.0)  L-9 (45.0) | R-11 (52.0)  L-10 (48.0) |
|  | **VAS (0-100)** | 63.7 (19.8) | 61.2 (21.2) |
|  | **Shoulder function (0-70)** | 39.3 (13.4) | 42.6 (14.5) |
|  |  |  |  |
| **Ucuncu 2009** |  |  |  |
|  | **Number** | 30 | 30 |
|  | **Age** | 52.9 (9.7) | 52.1 (11.6) |
|  | **% Female** | 22 (73.3) | 22 (73.3) |
|  | **Duration of symptoms** | 9.6 (8.7) months | 10.7 (12.5) months |
|  | **Affected shoulder** | R-18 (60.0)  L-12 (40.0) | R-13 (43.3)  L-17 (56.7) |
|  | **VAS (0-10)** | 6.0 (1.4) | 6.3 (1.8) |
|  | **Shoulder function (0-100)** | 70 (16.4) | 56.7 (21.6) |

*Mean (SD) reported

NSAID=non-steroidal anti-inflammatory drug; SASD=subacromial-subdeltoid; LMG=landmark-guided; USG=ultrasound-guided; US=ultrasound; ROM=range of movement; VAS=visual analogue scale; SFA=shoulder function assessment; R=right; L=left
